# Internet Sales of "Research Grade Compounds": The Opioid SR-17018 (SR-17) as an Example

**DOI:** 10.7759/cureus.116383

**Published:** 2026-09-16

**Authors:** Robert B Raffa, Joseph V Pergolizzi, Eugene Vortsman

**Affiliations:** 1 School of Pharmacy, Temple University, Philadelphia, USA; 2 Anesthesiology Department, NEMA Research, Naples, USA; 3 Emergency Department, Northwell, New Hyde Park, USA

**Keywords:** biased ligand, drug overdose, emergency medicine, internet drug sales, research grade compounds, sr-17, sr-17018

## Abstract

Several internet sites promote the sale of "research grade compounds." Although the purchaser attests that they are over 18 years of age, they know that the substances have not been approved by regulatory agencies and promise that they will not use them for human consumption, the compounds are supplied in tablet form, which is inconvenient for research use but very convenient for human consumption. One such compound is the opioid SR-17018 (aka "SR-17"). SR-17018 was originally synthesized at Scripps Research as part of a discovery effort to test whether a G-protein-‘biased’ mu-opioid receptor (MOR) agonist could separate the undesirable opioid effect of respiratory depression from the desirable effect of analgesia. It never progressed through the stages necessary for human clinical trials, so it remained a research "tool" drug. Regardless of the initial intent, the United States Drug Enforcement Agency (DEA) alerts that some "research grade compounds" such as SR-17018 are emerging as recreational or abused substances. If so, the absence of adequate human data creates a problem for healthcare providers who may encounter an overdose of such a compound without an adequate evidence base upon which to draw. This narrative review presents SR-17018 as a case study of the clinical uncertainty that a "research grade compound" introduces. It synthesizes in vitro, ex vivo, and plausible human pharmacology of SR-17018, and it presents an emergency department physician’s experience with a suspected SR-17 overdose.

## Introduction and background

Because the transduction of mu-opioid receptor (MOR) activation signals through both G protein and β-arrestin-2 second-messenger pathways, and because β-arrestin-2 recruitment was hypothesized to drive respiratory depression and tolerance, whereas G protein signaling was hypothesized to drive analgesia [1-3], a generation of "biased" (G protein-favoring) agonist compounds was synthesized to test whether they would lead to safer opioid analgesics [4-7]. SR-17018 emerged from such a discovery program. It is a functional agonist in G protein-coupled assays, but lacks β-arrestin-2 recruitment except at very high concentrations [4,8]. Like a number of drug discovery compounds, SR-17018 did not progress to clinical trials (so it is not approved for any medical use anywhere in the world), but it turned out to be a useful "tool" compound, informing research progress [4,9]. However, unlike most tool compounds, SR-17018 has suddenly (since 2024) become available for purchase through online sources as a "research grade compound," potentially as a recreational or abused substance [10,11]. At the time of writing, the regulatory status is adapting and is in flux: although it was not previously specifically scheduled in the United States, the Drug Enforcement Agency (DEA) emergency order of July 1, 2026 treats it as a Schedule I substance because of its structural and pharmacological similarity to controlled opioids; there is no European Union (EU)-wide specific scheduling, though it is monitored as a class substance; in May 2026 the United Kingdom (UK) recommended Class A scheduling for the entire class; and although Australia, Canada, and Japan have no specific scheduling of it currently, general analog or generic provisions likely apply. Such a situation mirrors that of other novel synthetic opioids (e.g., the nitazene class [5-7]), which moved from laboratory curiosities to street-level problems [12]. The purpose of this narrative review is to consolidate what is known about SR-17018's basic science pharmacology, what the preclinical pharmacology might predict for human use and overdose, and to provide an emergency department (ED) clinician’s presumptive treatment encounter.

## Review

Methodology

MEDLINE/PubMed, Embase, Scopus, Web of Science, preprint servers (bioRxiv, ChemRxiv), and the Cochrane Library were searched through August 2026 with no language restriction. Search terms included "SR-17018," "SR-17,” "5,6-dichloro-desmethylchlorphine," "biased agonis*," "G protein biased" AND "mu-opioid," "β-arrestin"-sparing agonist, "functionally selective" AND opioid SR-14968, SR-17018 AND (misuse OR abuse OR diversion OR "novel psychoactive substance" OR NPS OR "research chemical" OR "designer drug" OR overdose OR withdrawal OR taper) SR-17018 AND (clinical trial OR "phase I" OR "phase 1" OR human). We searched ClinicalTrials.gov, the WHO International Clinical Trials Registry Platform (ICTRP), the EU Clinical Trials Register, and the ISRCTN registry, plus Drugs@FDA, FDA Adverse Event Reporting System (FAERS), and EMA databases for any Investigational New Drug (IND) or marketing-authorization filings referencing SR-17018. No completed, ongoing, or terminated formally registered human clinical trial of SR-17018 was identified. We also searched the DEA National Forensic Laboratory Information System (NFLIS); DEA Federal Register notices and Diversion Control Division publications (including emergency or temporary scheduling actions and associated dockets); the UNODC Early Warning Advisory on NPS; the EMCDDA Early Warning System; the National Poison Data System (NPDS)/America's Poison Centers publications; and FAERS. In addition, we searched the open web and social media, including Google, Google Trends, Bing, Bluelight, Reddit subforums oriented toward opioid tapering or harm reduction, Erowid, vendor and research-chemical marketplace listings, news media, and advocacy-policy publications.

Basic (preclinical) pharmacology

**Figure 1:**
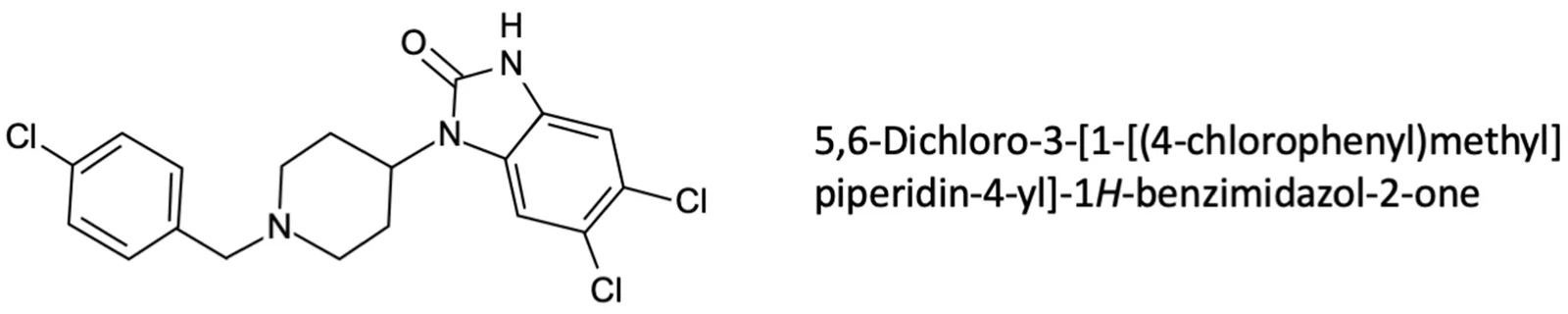
Chemical structure of SR-17018 (SR-17) Image credit: The authors using Microsoft PowerPoint (Microsoft, Redmond, WA, USA).

SR-17018 (5,6-Dichloro-3-[1-[(4-chlorophenyl)methyl]piperidin-4-yl]-1*H*-benzimidazol-2-one) (Figure 1) is an agonist at the MOR in G protein-coupled assays, such as GTPγS binding and inhibition of forskolin-stimulated cAMP accumulation, with potency in the low-to-mid nanomolar range [4]. In contrast, it has negligible efficacy for β-arrestin-2 recruitment in standard enzyme-fragment-complementation assays, other than at supra-therapeutic (micromolar) concentrations [4]. This pattern established SR-17018 among the most strongly G protein-biased compounds identified in the chemical series, together with SR-15098 and SR-15099, as distinguished from unbiased or arrestin-preferring reference opioids such as fentanyl and sufentanil in the same assay panels [4,13]. In the original in vivo characterization, these G protein-biased compounds produced antinociception (animal model of analgesia) with a wider separation from respiratory-depressant effects than did conventional opioids [4], which was the pharmacologic motivation for pursuing the compound as a potentially safer opioid analgesic scaffold.

Subsequent research has substantially complicated the simple bias viewpoint. A large multi-laboratory reanalysis concluded that the improved separation between analgesia and side effects seen with G protein-biased opioids, including compounds structurally related to SR-17018, is better explained by their low intrinsic efficacy at the receptor than by genuine pathway-selective (biased) signaling [9,14-20]. In other words, SR-17018 may behave more like a low-efficacy partial agonist under physiological receptor reserve conditions than like a pathway-selective “smart” opioid (the reason for the discrepancy from the in vitro assays, perhaps differences in assay system, receptor reserve, second-messenger transduction disparities, or other experimental conditions, is not known), a distinction with direct relevance for overdose risk. Receptor-level studies have also identified an unusual, biphasic phosphorylation signature for SR-17018 that is not consistent with the standard straightforward biased-ligand model. Although short-term exposures produce a phosphorylation pattern typical of a low-efficacy agonist, exposures beyond roughly 30 minutes produce a phosphorylation pattern indistinguishable from that of the full agonist peptide D-Ala^2^,*N*-MePhe^4^,Gly-ol enkephalin (DAMGO), but dephosphorylation occurs much more slowly than that induced by DAMGO [21]. Despite this atypical persistent receptor engagement, SR-17018-induced receptor phosphorylation remains fully reversible by naloxone during washout [21], a potentially important factor for overdose management. As a caveat, however, the efficacy, dose requirement, or duration of naloxone treatment in humans may not translate from nonclinical studies. Likewise, receptor dephosphorylation kinetics do not necessarily reflect receptor occupancy, plasma half-life, brain exposure, or duration of respiratory depression. Nevertheless, SR-17018 can be distinguished from typical full agonists, and this is central to why it drew scientific interest [22,23]. For example, in preclinical models, SR-17018 reverses established morphine tolerance and prevents opioid withdrawal signs in mice [24], a property not observed with other biased agonists tested in parallel and previously described only for buprenorphine among MOR ligands [25]. This anti-withdrawal, anti-tolerance profile has been attributed to SR-17018's atypical, delayed dephosphorylation kinetics at the receptor rather than to signaling bias per se [21]. Further study of this and other aspects of SR-17018's's intriguing pharmacology remains to be elucidated.

Current evidence and knowledge gaps regarding the clinical pharmacology of SR-17018

Some non-human primate data complicate simple extrapolation of the in vitro and rodent data to humans. Comparative studies of putative G protein-biased MOR agonists, including SR-17018, in rhesus monkeys found that the favorable separation between analgesia and respiratory depression seen in rodents was not consistently reproduced in non-human primates [26], underscoring that rodent therapeutic-window data for this compound cannot be assumed to generalize across species, let alone to humans.

Published animal pharmacokinetic data indicate that SR-17018 crosses the blood-brain barrier and is detectable in brain tissue after systemic administration, consistent with central MOR engagement, with plasma and brain exposure sufficient to produce measurable antinociceptive and physiological effects following peripheral dosing in rodent models [4]. Since regulatory approval was not pursued, human pharmacokinetic parameters are not published (e.g., bioavailability by any route, elimination half-life, protein binding, metabolic pathway, or drug-drug interaction potential), and no peer-reviewed human clinical trial, cohort study, or case series describing SR-17018 use, effects, or adverse events currently exists in the literature identified for this review. Consequently, any statement about onset, duration, or half-life in humans is necessarily inferential and should be treated as hypothesis-generating only. What follows is therefore a pharmacologically reasoned projection, not a synthesis of clinical observation, and should be labeled as such in any clinical communication about this substance.

Based on its action as an MOR agonist with clinically relevant in vivo potency, expected clinical effects of nonmedical SR-17018 use would plausibly include the standard opioid effect profile: analgesia, sedation, euphoria, miosis, and dose-dependent respiratory depression, along with gastrointestinal effects such as nausea and constipation typical of MOR activation. Because independent reanalysis attributes SR-17018's improved preclinical safety margin to low intrinsic efficacy rather than to a genuinely β-arrestin-sparing mechanism [9], low intrinsic efficacy agonists might have a ceiling effect on receptor activation analogous to that seen with agonists such as buprenorphine. But clinicians, and (ab)users, should not assume SR-17018 is inherently "safer" than full agonists at recreational doses, particularly given the primate data suggesting that the rodent therapeutic window does not reliably translate to other species [26].

The compound's unusual, persistent receptor engagement and slow dephosphorylation kinetics [21] raise the theoretical possibility of a prolonged pharmacodynamic effect at the receptor relative to its measured plasma exposure, and a correspondingly atypical time course of intoxication or re-sedation after apparent clinical improvement, though this has only been characterized in cellular systems and has not been confirmed as a clinically observable phenomenon in intact organisms or humans. This possibility nonetheless argues for cautious, extended observation in any suspected human exposure. Because SR-17018 has also been promoted informally as a withdrawal-mitigating agent, clinicians should consider that patients presenting after SR-17018 use may have a recent history of concurrent opioid use disorder, polysubstance use, or attempted self-directed opioid tapering, which independently affects risk stratification, co-ingestant screening, and disposition planning.

Overdose considerations

As of this date, no published human overdose case has been reported for SR-17018, so this section describes a rational toxidrome framework rather than documented outcomes. The core presumption, based on the receptor and cellular level, is that SR-17018 toxicity is MOR-mediated and naloxone-reversible, but this might not translate to humans. In vitro, SR-17018-induced receptor phosphorylation was fully reversed by naloxone during washout, despite the compound's atypically slow dephosphorylation kinetics under agonist exposure alone [21], supporting the expectation that naloxone could be the presumptive appropriate reversal agent for suspected SR-17018 toxicity, as it is for essentially all MOR agonists regardless of bias profile. But actual human validation data are lacking.

Two receptor-level features nonetheless warrant caution in overdose management, framed here explicitly as hypotheses rather than established clinical facts:

Possible need for repeat or extended naloxone dosing. If SR-17018's slow dephosphorylation and receptor engagement in cellular assays correspond to a longer functional receptor occupancy in vivo than would be predicted from plasma concentration alone, a single standard naloxone bolus could be followed by re-emergent sedation or respiratory depression, since naloxone (itself short-acting) wanes before the parent compound's receptor effect resolves, a pattern already well described for other long-acting or high-potency synthetic opioids and one that argues for observation and repeat dosing thresholds rather than reliance on a single-dose [27,28].

Low-efficacy/partial-agonist-like ceiling. If the compound's rodent (but not non-human primate) preclinical safety margin reflects low intrinsic efficacy rather than genuine pathway bias, as the reanalysis literature suggests [9], this would predict a ceiling on MOR-mediated respiratory depression analogous to buprenorphine, which could partially limit, but not eliminate, overdose severity, especially in opioid-naïve individuals or when in combination with other central nervous system (CNS) depressants. Such a ceiling effect, if it does exist in humans, would not protect against toxicity from co-ingested full agonists, benzodiazepines, or alcohol, which are common in unregulated drug supplies and in withdrawal self-management contexts.

The anecdotal observations of one ED physician in New York City (author EV) regarding the suspected effects of high-dose exposure to SR-17018 (unvalidated, but based on the patient’s self-report of the website source) are described below:

A patient presented to our clinic seeking standard treatment for opioid withdrawal and craving management. He was trying to taper himself off opioids and, following guidance he found on online forums, had turned to (presumptive) ‘SR-17’ as a self-directed withdrawal aid. Rather than bridging him off opioids, it became the substance he was now dependent on, and he arrived asking for conventional care. None of us treating him had any prior clinical experience with the substance he named.

That encounter reveals the practical dilemma that the ready availability of ‘research grade compounds’ creates. If it were indeed SR-17018 or other research compound, we were asked to manage withdrawal and craving for a compound with no published human pharmacokinetics, no established withdrawal timeline, and no evidence-base for how it responds to buprenorphine or other standard medications for opioid use disorder. We also had no way to know what he had actually taken. The product had been purchased online as a "research chemical," with no verification of its purity, content, dose, or quality, and no assurance it was free of more potent adulterants such as fentanyl or a nitazene. In effect, we were treating dependence on an unquantified substance of unknown composition, using protocols validated for entirely different drugs.

Emergency department management

Given the absence of validated human dosing data, there is no established toxic or lethal dose threshold for SR-17018, and illicitly obtained substances are not traditionally dosed or do not have a substantiated dosage in each product, and clinicians should not attempt to estimate from animal data, given the primate discordance noted above and inconsistent cross-species translation for the therapeutic separation between analgesic and respiratory effects of this compound class [26]. In the absence of a compound-specific treatment protocol, ED management of suspected SR-17018 exposure should follow the standard approach to an unknown or novel synthetic opioid toxidrome. It should be reiterated, though, that these are plausible but unconfirmed clinical considerations extrapolated from management of other opioids.

Recognition and Screening

A novel synthetic opioid, including SR-17018, should be suspected in a patient with an opioid toxidrome (e.g., altered mental status, miosis, respiratory depression) with a negative or inconsistent standard urine opioid immunoassay, since immunoassays do not detect most novel synthetic opioids and a negative screen does not exclude MOR agonist toxicity. Thus, a careful substance-use history should be obtained, including online or "research chemical" sourcing, including co-ingestants such as benzodiazepines, alcohol, stimulants, and other opioids, which materially change risk.

Airway, Breathing, and Circulation

Standard resuscitative priorities apply: supplemental oxygen, bag-valve-mask ventilation as needed, and continuous pulse oximetry-capnography monitoring for respiratory depression, which remains the dominant life threat with any MOR agonist.

Naloxone Administration

Naloxone remains the presumptive reversal agent, given the receptor-level evidence that SR-17018 effects are naloxone-reversible. Titration using incremental dosing to effective spontaneous ventilation rather than full arousal is desired to avoid precipitating acute withdrawal in patients with underlying opioid dependence. Given the concern about a possibly prolonged receptor effect relative to naloxone's short half-life, clinicians should have a low threshold for repeat dosing or a naloxone infusion if re-sedation or recurrent respiratory depression occurs, and should extend observation time beyond what might usually be adequate for a short-acting conventional opioid.

Observation Duration

In the absence of validated human elimination data, an extended ED observation period (analogous to protocols used for long-acting or high-potency synthetic opioid exposures) is a reasonable precaution, and specific observation duration has not been established, with disposition decisions based on sustained resolution of respiratory depression and mental status rather than a fixed cutoff time.

Supportive Care

Management of nausea and monitoring for aspiration risk in obtunded patients are desirable. There is no antidote or supportive intervention specific to SR-17018 beyond standard opioid-overdose supportive care.

Reporting and Public Health

Given the compound's status as an unscheduled, unregulated substance now entering nonmedical use, suspected cases should be reported to regional poison control centers and, where available, to public health or forensic toxicology surveillance systems for novel psychoactive substances, since confirmatory testing (liquid chromatography-tandem mass spectrometry) at a reference laboratory will typically be required to make a definitive identification; routine hospital toxicology panels do not detect SR-17018.

Behavioral Health and Harm Reduction

Because informal use of SR-17018 appears linked to self-directed opioid withdrawal management, any ED encounter is an opportunity to engage patients with substance use disorder treatment resources, including medication for opioid use disorder, given the possible underlying opioid dependence in this population.

Discussion and limitations

A summary of the current state of knowledge (what is known and not known) about the pharmacology and clinical effects of use and overdose of SR-17018 in humans is shown in Table 1.

**Table 1:** Current evidence and knowledge gaps relevant to the pharmacology and clinical management of SR-17018. MOR = mu-opioid receptor; PK = pharmacokinetics; CNS = central nervous system; OD = overdose; SR-17 = SR-17018.

Domain	What is known	Evidence	What is unknown
MOR activity	Established	In vitro	Human potency
G-protein signaling	Established	In vitro	Clinical relevance
β-arrestin recruitment	Minimal in assays	In vitro	Human relevance
Analgesia	Demonstrated	Rodents	Human analgesic effect
Respiratory depression	Preclinical findings	Animal	Human dose-response
PK	CNS penetration	Animals	Human half-life and metabolism
Naloxone reversibility	Receptor evidence	In vitro	Human dose requirements
Withdrawal effects	Preclinical	Animal	Human withdrawal phenotype
OD management	Extrapolated	Standard care	SR-17-specific protocol

This manuscript necessarily rests on an asymmetric evidence base: extensive (but by no means definitive, and even contradictory) in vitro and rodent in vivo pharmacology, markedly weaker and sometimes oppositional non-human-primate data, and an absence of controlled human data. This is precisely the situation that makes SR-17018 a clinically important topic despite, or rather because of, the lack of case reports: clinicians are likely to encounter unrecognized exposures before the peer-reviewed clinical literature catches up, a pattern seen repeatedly with prior classes of novel synthetic opioids. We have deliberately distinguished herein between statements that are grounded in cited experimental data and statements that are reasoned extrapolations; the latter should be treated as clinical hypotheses to guide caution, not as established fact, and should be revised as soon as real human case data become available. We did not find any published human case reports, cohort studies, or controlled trials of SR-17018 in the course of preparing this review, and the clinical report herein is anecdotal, so readers should independently verify that this remains the case at the time of publication given how rapidly this compound's non-medical use appears to be evolving.

## Conclusions

SR-17018 (SR-17) is a pharmacologically well-characterized preclinical MOR agonist with an atypical receptor engagement profile, without established human safety or efficacy data, that has moved from the laboratory into unregulated non-medical use faster than the clinical evidence base has developed. Until better information is available, emergency clinicians are left to treat suspected SR-17018 toxicity as they would treat any unidentified synthetic opioid exposure: aggressive airway support, naloxone titrated to effective ventilation with a low threshold for repeat dosing, extended observation given theoretical concerns about prolonged receptor engagement, and engagement with poison control, toxicology surveillance, and substance-abuse treatment resources. Formal human pharmacokinetic, toxicological, and clinical outcome data are needed if this compound's non-medical use expands.
